# Lithia/(Ir, Li_2_IrO_3_) nanocomposites for new cathode materials based on pure anionic redox reaction

**DOI:** 10.1038/s41598-019-49806-6

**Published:** 2019-09-12

**Authors:** Si Yeol Lee, Yong Joon Park

**Affiliations:** Department of Advanced Materials Engineering, Graduate School Kyonggi University, 154-42, Gwanggyosan-Ro, Yeongtong-Gu, Suwon-Si, Gyeonggi-Do 16227 Republic of Korea

**Keywords:** Batteries, Batteries

## Abstract

Anionic redox reactions attributed to oxygen have attracted much attention as a new approach to overcoming the energy-density limits of cathode materials. Several oxides have been suggested as new cathode materials with high capacities based on anionic (oxygen) redox reactions. Although most still have a large portion of their capacity based on the cationic redox reaction, lithia-based cathodes present high capacities that are purely dependent upon oxygen redox. Contrary to Li-air batteries, other systems using pure oxygen redox reactions, lithia-based cathodes charge and discharge without a phase transition between gas and condensed forms. This leads to a more stable cyclic performance and lower overpotential compared with those of Li-air systems. However, to activate nanolithia and stabilize reaction products such as Li_2_O_2_ during cycling, lithia-based cathodes demand efficient catalysts (dopants). In this study, Ir based materials (Ir and Li_2_IrO_3_) were introduced as catalysts (dopants) for nanolithia composites. Oxide types (Li_2_IrO_3_) were used as source materials of catalyst because ductile metal (Ir) can hardly be pulverized during the milling process. Two types of Li_2_IrO_3_ were prepared and used for catalyst-sources. They were named ‘1-step Li_2_IrO_3_’ and ‘2-step Li_2_IrO_3_’, respectively, since they were prepared by ‘1-step’ or ‘2-step’ heat treatment. The nanocomposites prepared using lithia & 2-step Li_2_IrO_3_ presented a higher capacity, more stable cyclic performance, and lower overpotential than those of the nanocomposites prepared using lithia & 1-step Li_2_IrO_3_. The voltage profiles of the nanocomposites prepared using lithia & 2-step Li_2_IrO_3_ were stable up to a limited capacity of 600 mAh·g^−1^, and the capacity was maintained during 100 cycles. XPS analysis confirmed that the capacity of our lithia-based compounds is attributable to the oxygen redox reaction, whereas the cationic redox related to the Ir barely contributes to their discharge capacity.

## Introduction

Nowadays, lithium ion batteries (LIBs) are widely used as power sources in various applications such as portable electronics, electric vehicles, and energy storage systems. As these technologies become more complex, they require advanced LIBs with higher energy densities without exception, which necessitates new cathode materials. In the past four decades, many materials based on transition metal oxides have been explored as potential improved cathode materials with superior energy densities to conventional cathodes such as LiCoO_2_^1–5^. As a result, the Li(Ni, Co, Mn)O_2_, Li(Ni, Co, Al)O_2_, and Li(Li, Ni, Mn)O_2_ (over-lithiated oxides) groups have been suggested as high-capacity cathode materials^6–10^. These materials have contributed to the improved energy densities of LIBs; however, they are now thought to be nearing their capacity limits.

Recently, the new concept of cathode materials based on anionic (oxygen) redox reactions has been explored to overcome reversible capacity limitations. Fundamentally, the reversible capacities of the above-mentioned cathode materials are based only on the ‘cation redox reaction’ associated with transition metals in the oxides. In contrast, several materials such as Li-Nb-Mn-O, Li-Mn-O, and Li-Ru-M-O (M = Sn, Nb) have taken advantage of combining both cation and anion (oxygen) redox reactions within the same compound, which have yielded high capacities exceeding 300 mAh·g^−1^ ^11–14^. However, these materials have several issues, such as capacity fading and sluggish kinetics attributed to the anion redox process. On the other hand, Li-air batteries, which are based on a ‘pure’ oxygen redox reaction, exhibit energy densities far exceeding those achievable by LIB systems because light oxygen is used as the cathode material instead of heavy transition metal oxides. However, their commercialization has been seriously limited by several major challenges including limited cyclic performance and high overpotentials. These challenges are inherently linked to the redox system based on a phase change from gaseous oxygen to a condensed phase (Li_2_O_2_)^15–20^.

In this respect, nanolithia (Li_2_O)-based materials could be promising cathode materials based on pure oxygen redox reactions^21–25^. Their reversible capacities are based on the oxygen redox reaction between O^2−^ (Li_2_O) and O_2_^2−^ (Li_2_O_2_), which could provide a high theoretical capacity (897 mAh·g^−1^). Moreover, they have shown stable cyclic performances and low overpotentials because the redox reaction occurs within the solid phase without a phase transition, unlike in Li-air systems. However, the activation of capacities of nanolithia-based materials are highly dependent upon doping ions acting as catalysts^22–25^. Furthermore, their cyclic performances and rate capabilities are also highly sensitive to the composition and amount of doping material. Several doping materials such as Co_3_O_4_^22^, LiFeO_2_^23^, LiCoO_2_^24^, and CuO^25^ have been suggested to activate nanolithia and stabilize reaction products like Li_2_O_2_. However, the research on efficient catalysts for activating nanolithia remains in its infancy.

In the present study, new catalysts (doping materials) based on Ir are introduced to enhance the capacity and stability of nanolithia-based materials. Ir is a typical catalytic material, but in its metallic state, the ductile metal can hardly be pulverized and mixed with nanolithia during the milling process for preparing nanolithia-based composites. Instead, Ir oxides containing Li ions (Li_2_IrO_3_) were chosen as catalysts-sources in this work. The oxides are expected to be easily pulverized and reacted with the nanolithia through milling. In this process, metallic Ir having a high catalytic activity could be formed. Moreover, the oxide dopants containing Li (such as LiCoO_2_) have acted as better catalysts than binary oxides (such as Co_2_O_3_) because they are advantageous for homogeneous doping and have more vacancies for lithium ion movement^24^. Furthermore, Ir oxides has been reported to promote anion redox reactions^13,26,27^.

We focused our research on obtaining a nanolithia-based cathode with a good capacity and cyclic performance. The effect of an Ir-based catalyst on nanolithia activation was observed using X-ray diffraction (XRD), X-ray photoelectron spectroscopy (XPS), and electrochemical measurements.

### Comparison of two types of nanocomposites

**Figure 1:**
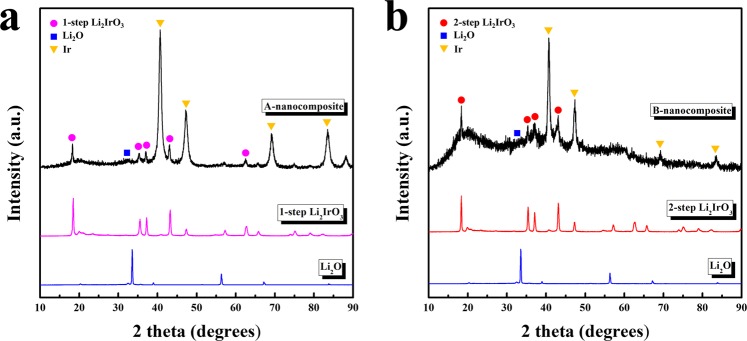
XRD patterns of the nanocomposites compared with 1-step and 2-step Li_2_IrO_3_ and lithia (Li_2_O): (**a**) A-nanocomposite; (**b**) B-nanocomposite.

Two types of Li_2_IrO_3_ were used as catalyst-souce for the lithia-based nanocomposites. They were named ‘1-step Li_2_IrO_3_’ and ‘2-step Li_2_IrO_3_’, respectively, since they were prepared by ‘1-step’ or ‘2-step’ heat treatment. The lithia and two-types of Li_2_IrO_3_ were reacted using milling process to form nanocomposites, and structural and electrochemical properties of them were investigated. Hereafter two types of nanocomposites prepared using ‘lithia & 1-step Li_2_IrO_3_’ or ‘lithia & 2-step Li_2_IrO_3_’ denoted as A-nanocomposite and B-nanocomposite, respectively. Figure 1 shows the XRD patterns of the A- and B-nanocomposites. The diffraction patterns of the 1-step and 2-step Li_2_IrO_3_ and Li_2_O (raw materials) are also included at the bottom of the figure. As shown in Fig. 1a, the A-nanocomposite pattern was composed of large Ir peaks and small Li_2_IrO_3_ peaks. The large Ir peaks indicated that a considerable amount of Li_2_IrO_3_ was decomposed to Ir during the milling process, although a small portion of Li_2_IrO_3_ seemed to retain its original form. The low intensity of the broad LiO_2_ peaks suggested that most of the crystalline lithia was converted to amorphous phase during milling. This decrease in the crystallinity of lithia by the milling process has been reported previously^22–25^.

As shown in Fig. 1b, the XRD pattern of the B-nanocomposite was somewhat different from that of the A-nanocomposite. Although large Ir peaks suggesting partial decomposition of crystalline Li_2_IrO_3_ were present, the broad and relatively large peaks associated with Li_2_IrO_3_ indicated that a considerable portion of crystalline Li_2_IrO_3_ changed to an amorphous-like phase without decomposition. The 2-step Li_2_IrO_3_ is more stable than 1-step Li_2_IrO_3_ because it has been heat-treated for longer time. This leads to relatively larger amount of residual Li_2_IrO_3_ in the B-nanocomposite compared with that in the A-nanocomposite. The peaks related to crystalline lithia were almost non-existent as well. Considering these results, it is presumed that the B-nanocomposite mostly consisted of crystalline Ir, amorphous-like Li_2_IrO_3,_ and amorphous lithia. The amorphous lithia phase is expected to have a positive effect on the electrochemical properties of the resulting material, such as a reduction in overpotential because the energy required for amorphous phase transitions is relatively small compared with that required for crystalline phase transitions^21^.

**Figure 2:**
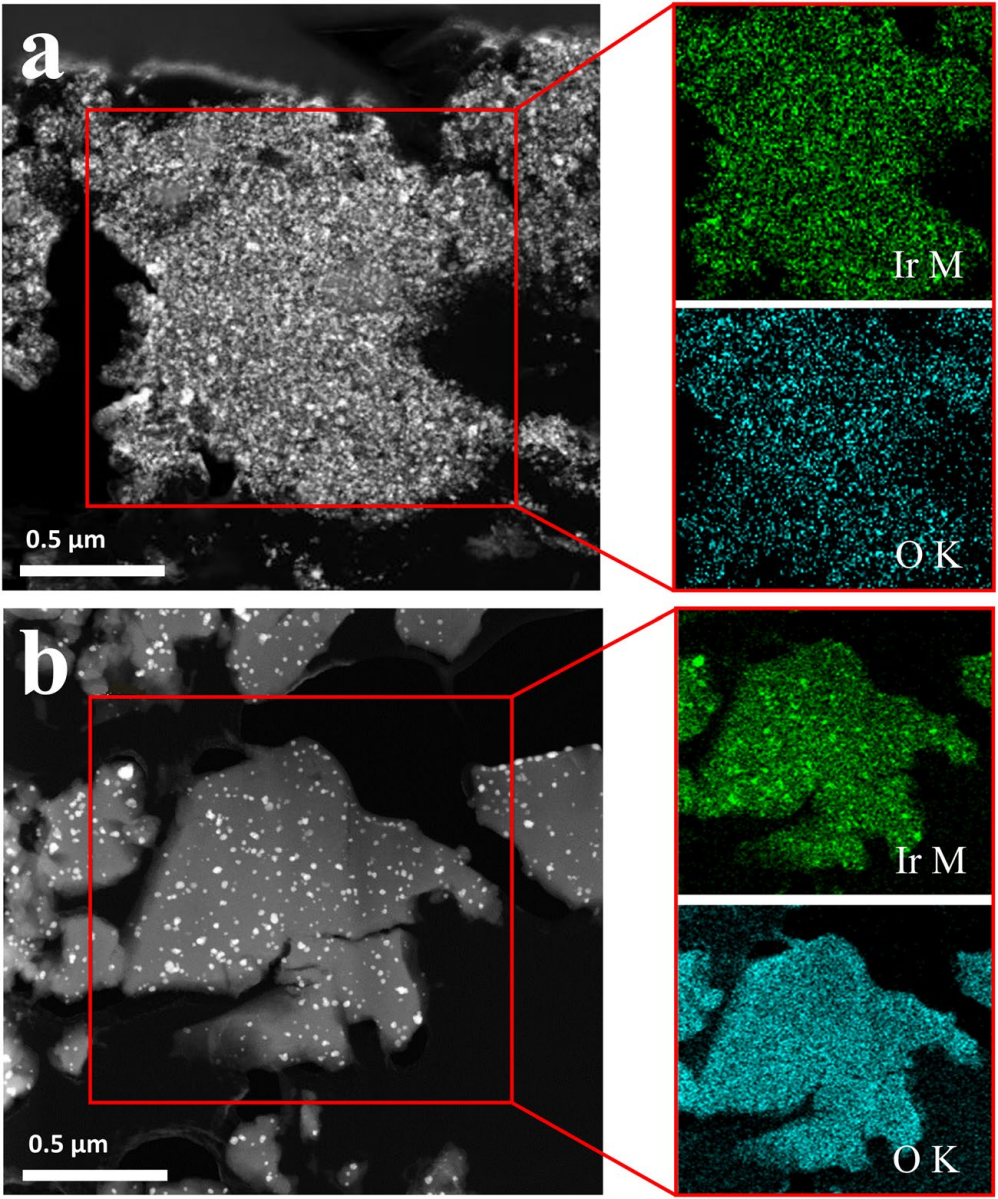
TEM images (left) and EDS elemental maps (right) of nanocomposite powders: (**a**) A-nanocomposite; (**b**) B-nanocomposite.

The distributions of Ir and Li_2_IrO_3_ may have a considerable effect on their catalytic activity in the nanocomposites. The structural morphologies of the nanocomposites were observed using TEM images and energy-dispersive X-ray spectroscopy (EDS) elemental mapping, as shown in Fig. 2. The bright spots in the TEM images seemed to be Ir particles, and the rest of the material would then be a mixture of Li_2_IrO_3_ and lithia. As shown in the EDS elemental maps, Ir and O were homogeneously distributed in the A- and B-nanocomposites, showing that they were composed of a uniform mixture of lithia, Ir, and Ir-oxides.

**Figure 3:**
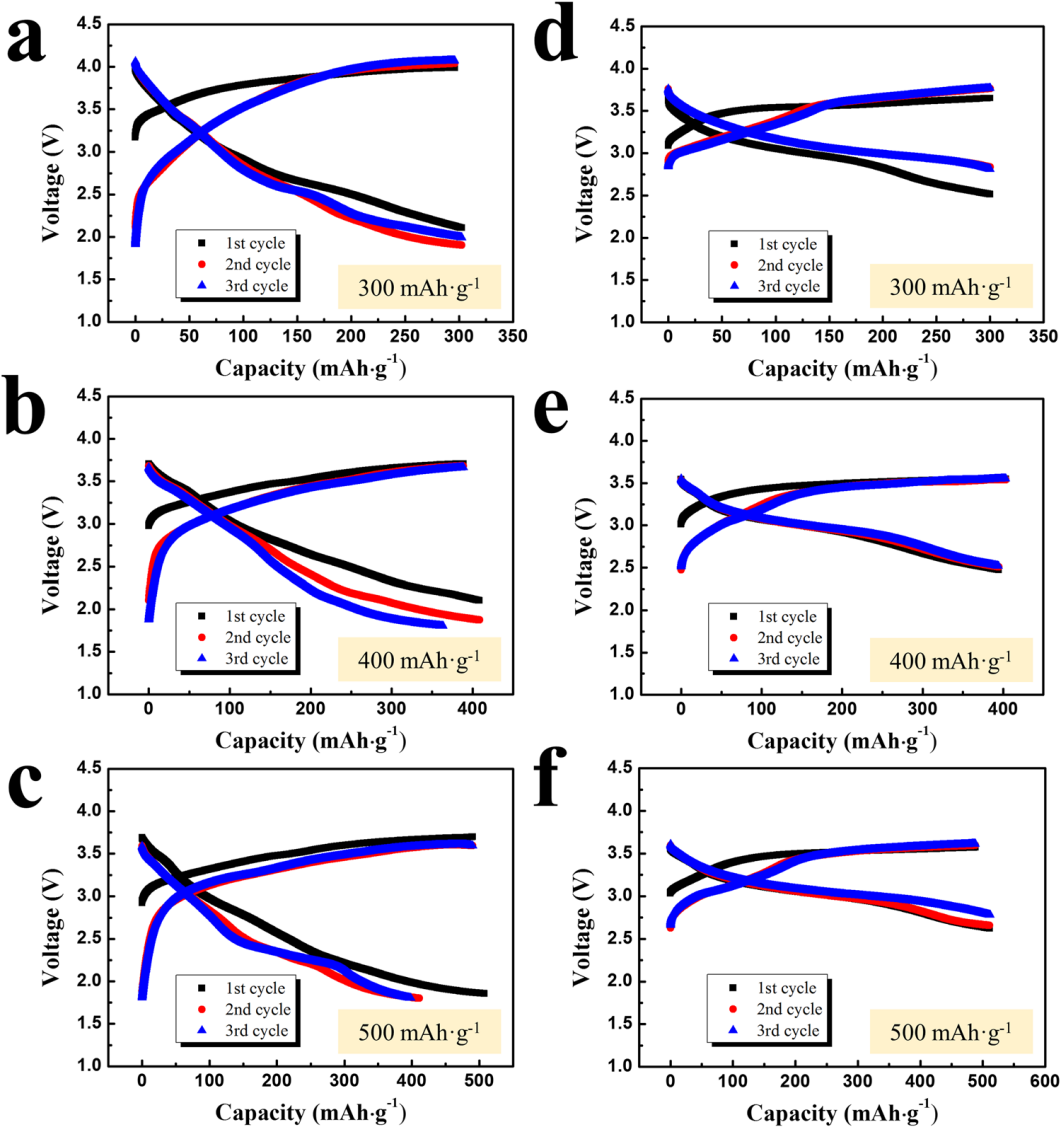
Voltage curves of nanocomposites at a current density of 10 mA·g^−1^. (**a**–**c**) A-nanocomposite, capacity limited to 300, 400, and 500 mAh·g^−1^, respectively; (**d**–**f**) B-nanocomposite, capacity limited to 300, 400, and 500 mAh·g^−1^, respectively.

To investigate the effects of Ir and Li_2_IrO_3_ doping and determine which type of Li_2_IrO_3_ was more effective as a catalyst-source, the electrochemical properties of the two types of nanocomposites were measured and compared. Figure 3 shows the voltage curves of the A- and B-nanocomposites at a current density of 10 mA·g^−1^. The charge capacity was limited to 300, 400, and 500 mAh·g^−1^ based on lithia weight to determine the stable capacity limits of the compounds, as over-charging of lithia-based compounds has been shown to cause a rapid decrease in capacity^22–25^. As shown in Fig. 3a, the voltage curves of the A-nanocomposite did not change greatly during 3 cycles when the capacity was limited to 300 mAh·g^−1^. However, as the limited charge capacity was increased to 400 and 500 mAh·g^−1^, the discharge capacity of the A-nanocomposite decreased distinctly (Fig. 3b,c) during cycling. The cyclic performance of the A-nanocomposite shown in Fig. S1a–c also indicated the instability of the A-nanocomposite during cycling at 400 and 500 mAh·g^−1^ limited capacities. In contrast, the cyclic performance of the B-nanocomposite was more stable than that of the A-nanocomposite. As shown in Fig. 3d–f, the discharge capacities and voltage profiles of the B-nanocomposite were stable during 3 cycles in the limited capacity range of 300–500 mAh·g^−1^. Figure S1d,f confirms the better cycle life of the B-nanocomposite for long cycles compared with that of the A-nanocomposite.

The rapid capacity fading of the A-nanocomposite may be attributable to the over-charging phenomenon because when lithia (Li_2_O) is charged above the limit to maintain a condensed phase, oxygen (O_2_) evolution can occur, which leads to irreversible capacity loss. Thus, the better cycle life of the B-nanocomposite (at high limited capacities) than that of the A-nanocomposite means that the use of 2-stpe Li_2_IrO_3_ is more effective for activating lithia (Li_2_O) and achieving higher capacities. More precisely, the Ir-based materials formed from 2-step Li_2_IrO_3_ are acting as superior catalysts than those formed from 1-step Li_2_IrO_3_.

The stabilization of reaction products in the nanocomposites is also an important factor for obtaining stable cyclic performances. Reaction products such as peroxides that form during the charging process are unstable and thus can easily react with the electrolyte, which also reduces capacity during cycling. Contact with dopant materials (catalysts) can stabilize these unstable reaction products and thereby stabilize the cyclic performance^21^. Thus, it is considered that unstable reaction products are stabilized more efficiently by the use of 2-step Li_2_IrO_3_ compared with the use of 1-step Li_2_IrO_3_. The smaller voltage difference of the B-nanocomposite between charge and discharge curves, indicating a lower overpotential, than that of the A-nanocomposite also supported the fact that the use of 2-step Li_2_IrO_3_ is superior to 1-step Li_2_IrO_3_ for preparing lithia-based composites. The lower overpotential shows that the energy consumption during the charging-discharging process is smaller, which further confirms the higher efficiency of the use of 2-step Li_2_IrO_3_ as a catalyst-source.

Actually, the improved electrochemical properties of the B-nanocomposite may not indicate that the catalytic activity of 2-step Li_2_IrO_3_ is higher than that of 1-step Li_2_IrO_3_ because their crystalline phase was decomposed and changed during the milling process. Considering the previous XRD analysis (Fig. 1), whereas 1-step Li_2_IrO_3_ seemed to be mostly decomposed to Ir during milling_,_ a considerable amount of 2-step Li_2_IrO_3_ appeared to maintain its oxide form as an amorphous phase. It is possible that this difference is related to the electrochemical performance of the resulting nanocomposites. Previous studies have reported that oxide dopants containing Li are effective as catalysts for lithia^22–25^. The relatively large amount of amorphous Li_2_IrO_3_ in the B-nanocomposite can act as an effective catalyst for activating lithia and stabilizing reaction products such as peroxides.

### Properties of B-nanocomposite

**Figure 4:**
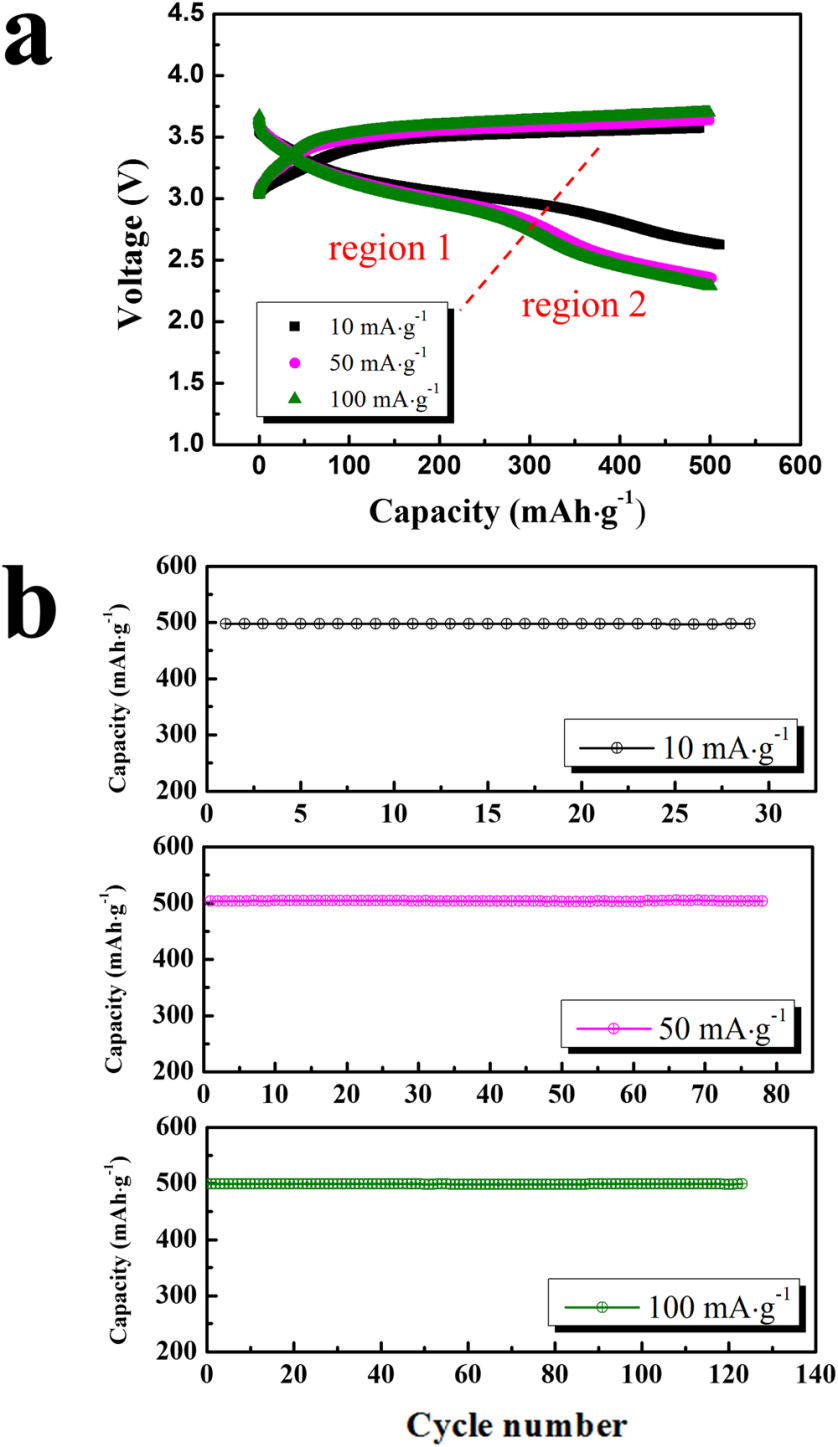
Voltage profiles and cyclic performance of the B-nanocomposite at current densities of 10, 50, and 100 mA·g^−1^ and limited capacity of 500 mAh·g^−1^: (**a**) voltage profiles, (**b**) cyclic performance.

The electrochemical performance of the B-nanocomposite was analysed in more detail. Figure 4a shows the voltage profiles of the B-nanocomposite at current densities of 10, 50, and 100 mA·g^−1^ with a limited capacity of 500 mAh·g^−1^. As the current density increased, the overpotential of the cells containing B-nanocomposite increased slightly. Notably, the low-voltage range of the discharge curves showed relatively large changes. The voltage curves at discharging could be divided into two regions. If the discharge capacity was fully generated from the oxygen redox reaction, the high-voltage range (marked as region 1) is related to the annihilation of peroxo-like (O_2_)^*n*−^ species formed during the charging process^24^. The low-voltage range (marked as region 2) is associated with the neutralization of O 2 P holes. However, the possibility that other redox reactions could have contributed to the full discharge capacity of the B-nanocomposite could not be excluded. In actuality, the discharge capacities generated from pure lithia have been reported in a low voltage range of less than 3.1–3.2 V. However, that of the B-nanocomposite started at a higher voltage of approximately 3.5 V in this work. Furthermore, Li_2_IrO_3_ itself can attribute to the capacity of the B-nanocomposite because it is a cathode material with its own considerable capacity. However, Li_2_IrO_3_ requires high-voltage charging (~4.8 V), and its discharging capacity is delivered at a higher voltage range (above ~3.5 V) than the voltage region observed in the B-nanocomposite. Thus, the majority of the discharge capacity of the B-nanocomposite may not be associated with the capacity of Li_2_IrO_3_. However, a small portion of the capacity at a high voltage range of approximately 3.2–3.6 V is debatably attributable to Li_2_IrO_3_ or other species formed from its decomposition. As shown in Fig. 4b, the cyclic performance of the B-nanocomposite was stable at high current densities (50 and 100 mA·g^−1^), showing its good rate capability.

The limited capacity for the B-nanocomposite was increased to 600 and 700 mAh·g^−1^ to identify the capacity limit for maintaining a stable cyclic performance. As shown in Fig. S2a,c, the B-nanocomposite showed a stable voltage profile at a limited capacity of 600 mAh·g^−1^. However, when the limited capacity was increased to 700 mAh·g^−1^, capacity fading during cycling clearly occurred (Fig. S2b). This capacity fading means that the B-nanocomposite was over-charged, which results in side reactions such as oxygen evolution. Shuttling inside the electrolyte can also occur in an over-charged state^21^. The super-oxoradicals formed during over-charging can diffuse to the anode and acquire electrons, then return to the anode, resulting in a shuttling of current through the electrolyte and leading to a lower discharge capacity than charge capacity. However, this shuttling process occurs in the plateau region, where a constant voltage is maintained in the charge curve, and such a region is not clearly visible in Fig. S2b. Therefore, the capacity fading of the B-nanocomposite at a limited capacity of 700 mAh·g^−1^ is not closely related to shuttling. Although the capacity should be limited to prevent over-charging phenomena, the cyclic performance of the B-nanocomposite (within the capacity limit of 600 mAh·g^−1^) seemed to be superior to that of previously reported lithia-based cathodes prepared by milling processes^22–25^. As shown in Fig. S2c, the capacity of 600 mAh·g^−1^ remained stable for 100 cycles.

**Figure 5:**
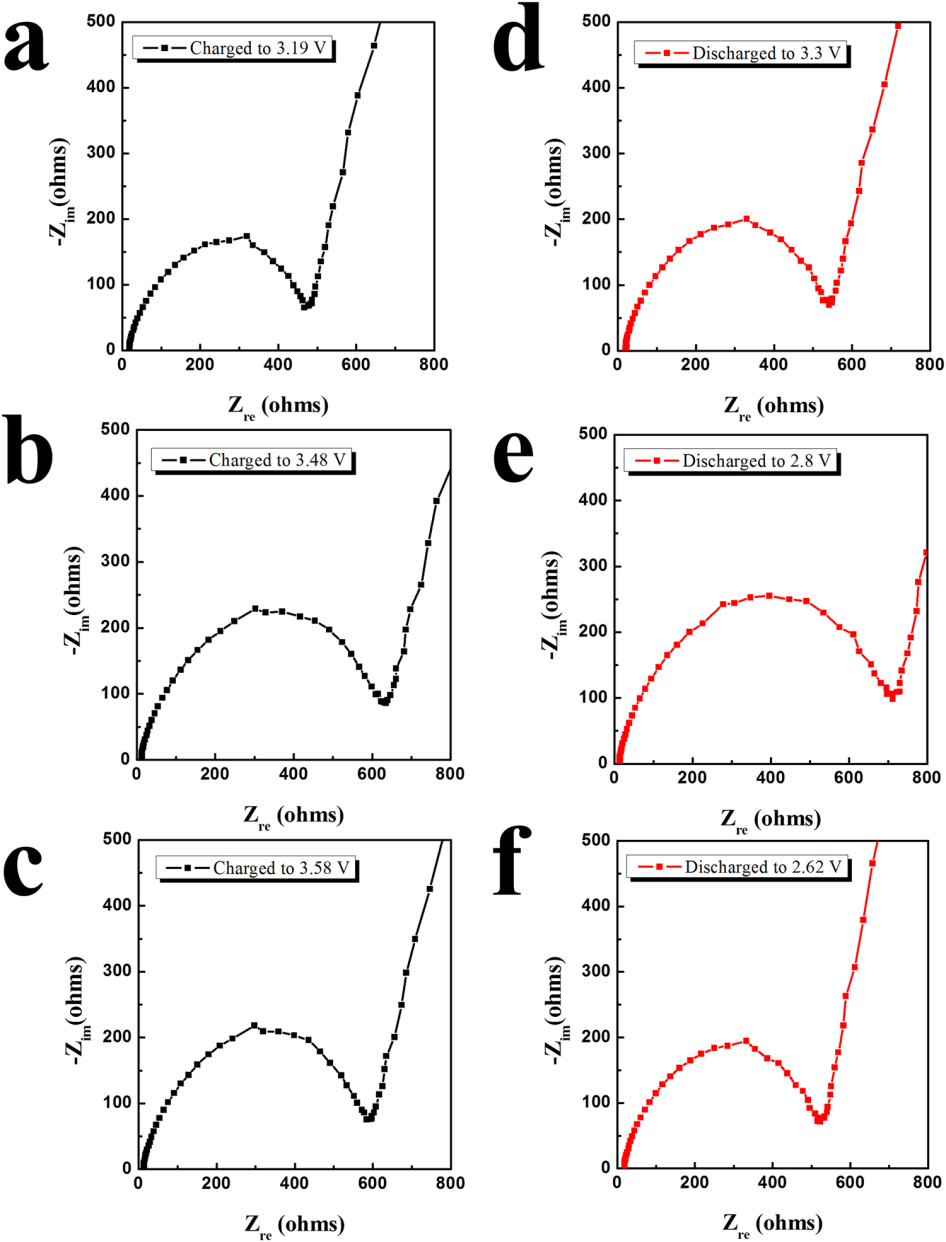
Nyquist plots of cell containing B-nanocomposite at the charging and discharging points marked in Fig. S3: Charged to (**a**) 3.19 V, (**b**) 3.48 V, and (**c**) 3.58 V; discharged to (**d**) 3.3 V, (**e**) 2.8 V, and (**f**) 2.62 V.

Figure S3 presents the corresponding derivative plots (d*Q*/d*V*) of the initial cycles in Fig. 3f. A broad peak at ~3.19 V and large peak at ~3.5 V were observed in the charging profile. In the discharging process, two broad peaks were present at approximately 3.0 and 2.6 V, which may be mainly related to the annihilation of peroxo-like (O_2_)^*n*−^ species and neutralization of O 2 P holes, respectively. The small peaks at approximately 3.4~3.5 V suggest that other redox reactions may participate in the charging-discharging process of the B-nanocomposite. The Nyquist plots of the cells at several charging and discharging points marked on Fig. S3 are shown in Fig. 5. A general Nyquist plot of lithium-ion cells is composed of two semicircles related to the charge transfer resistance and solid-electrolyte interface (SEI). However, all of the Nyquist plots in Fig. 5 seemed to be composed of one circle, which may due to the overlapping of two semicircles. It was difficult to separate the charge transfer resistance and SEI impedance from the plots, and obtain the fitting resistance value from the equivalent circuit due to severly overlapped semicircles. However, the increase and decrease in the impedance value can be analysed through the semicircle sizes. The size of the semicircle increased during charging to 3.48 V (Fig. 5a,b) and then remained almost the same until the cell was fully charged (Fig. 5c), indicating that the impedance value increased until it reached a specific charged state and then remained nearly the same. During discharging to 3.3 V, the size of the semicircle was highly similar to that of the fully charged semicircle (Fig. 5d). Interestingly, the semicircle size increased considerably during further discharging to 2.8 V (Fig. 5e), which suggests that the annihilation of peroxo-like (O_2_)^*n*−^ species may increase the impedance of the cell. The size decreased again until the cell was fully discharged (Fig. 5f), indicating that the neutralization of O 2 P holes reduced the impedance of the cell.

**Figure 6:**
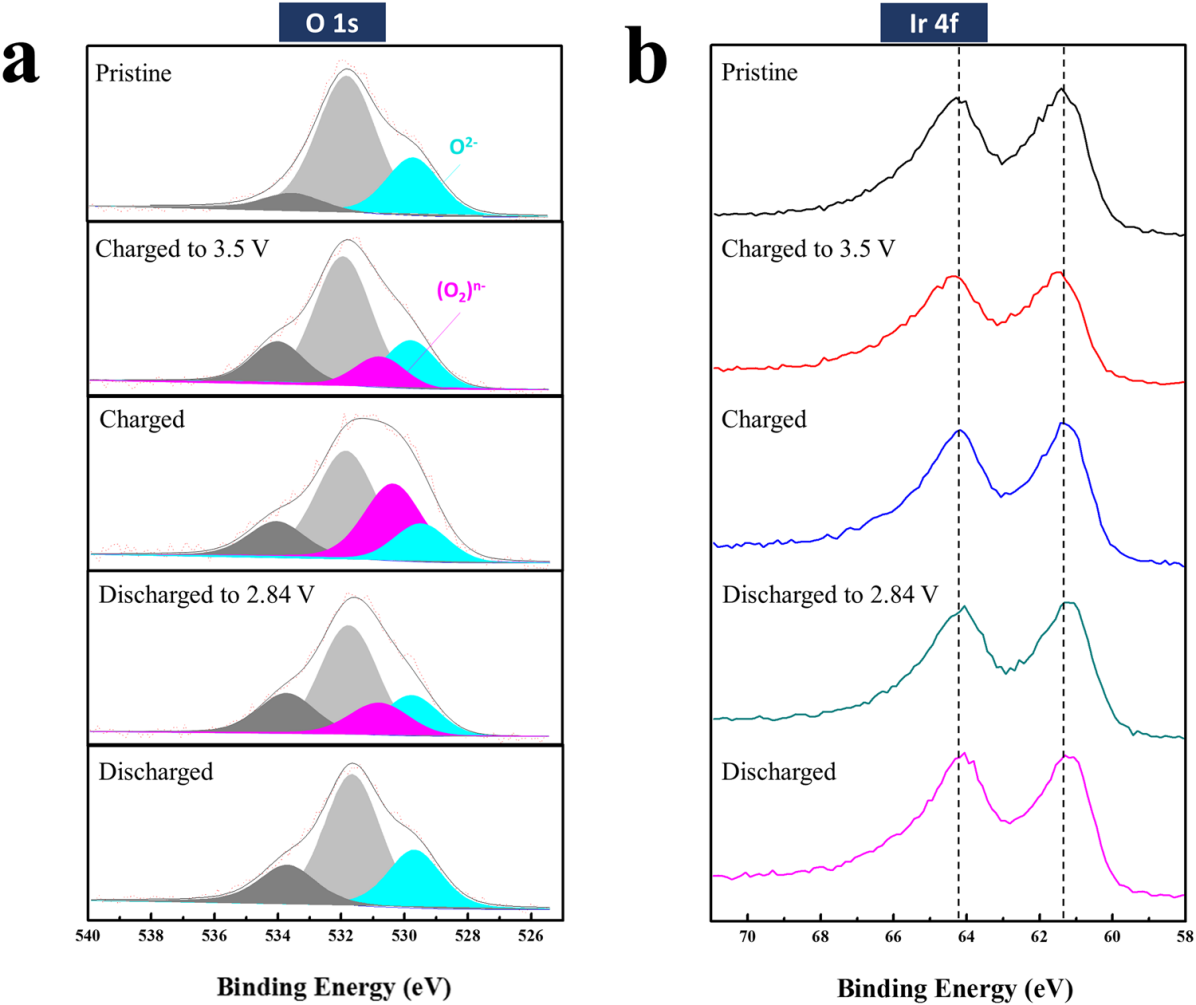
XPS spectra of the B-nanocomposite measured at various charging and discharging points: (**a**) O 1 s spectra, (**b**) Ir 4 f spectra.

To elucidate the redox reaction mechanism of the B-nanocomposite during the charging-discharging process, XPS measurements were carried out on pristine, *ex-situ* charged to 3.5 V, fully charged (as shown in Fig. S3), discharged to 2.84 V, and fully discharged samples. Figure 6a shows the O 1 s spectra of the B-nanocomposite during 1 cycle. In the charging process, a new peak at approximately 531 eV (marked in pink) appeared and grew until the cell was fully charged; this was attributed to the formation of peroxo-like (O_2_)^*n*−^ species through the oxygen redox reaction during the charging process. The peak related to peroxo-like species decreased during the discharging process and had almost disappeared once the cell was fully discharged. These results confirmed the reversible oxidation and reduction reaction of oxygen. In contrast, the Ir 4 f peak did not markedly change during the charging and discharging process, as shown in Fig. 6b. According to a previous report^26^, the capacity of Li_2_IrO_3_ is attributable not only to oxygen redox related to the peroxo-like species but also to the cationic redox reaction related to Ir. In the XPS analysis of Li_2_IrO_3_, a shift in the Ir 4 f peak was clearly observable during cycling^26^. However, the XPS spectrum of the B-nanocomposite did not show a meaningful 4 f peak shift, confirming that the cationic redox reaction did not contribute greatly to the capacity of the B-nanocomposite. As discussed above, it is possible that the capacity generated from amorphous Li_2_IrO_3_ contributed to the discharge capacity of the B-nanocomposite. Considering that the XPS results showed that a majority of the capacity was attributable to anionic (oxygen) redox reactions, the capacity generated from cationic redox reactions related to Ir may be negligible. However, the anion (oxygen) in the amorphous Li_2_IrO_3_ structure can participate in oxygen redox reactions, which accounts for a portion of the capacity. Moreover, the Li-O compounds formed from the decomposition of Li_2_IrO_3_ during the milling process can also contribute to oxygen redox reactions during cycling. The small capacity (less than 70–80 mAh·g^−1^) detected at a high voltage range (approximately 3.1–3.6 V), as shown in Fig. 4a, may be mainly associated with these other oxygen redox reactions. The remainder of the capacity is mostly attributable to the oxygen redox reaction of lithia in the B-nanocomposite.

## Summary

**Figure 7:**
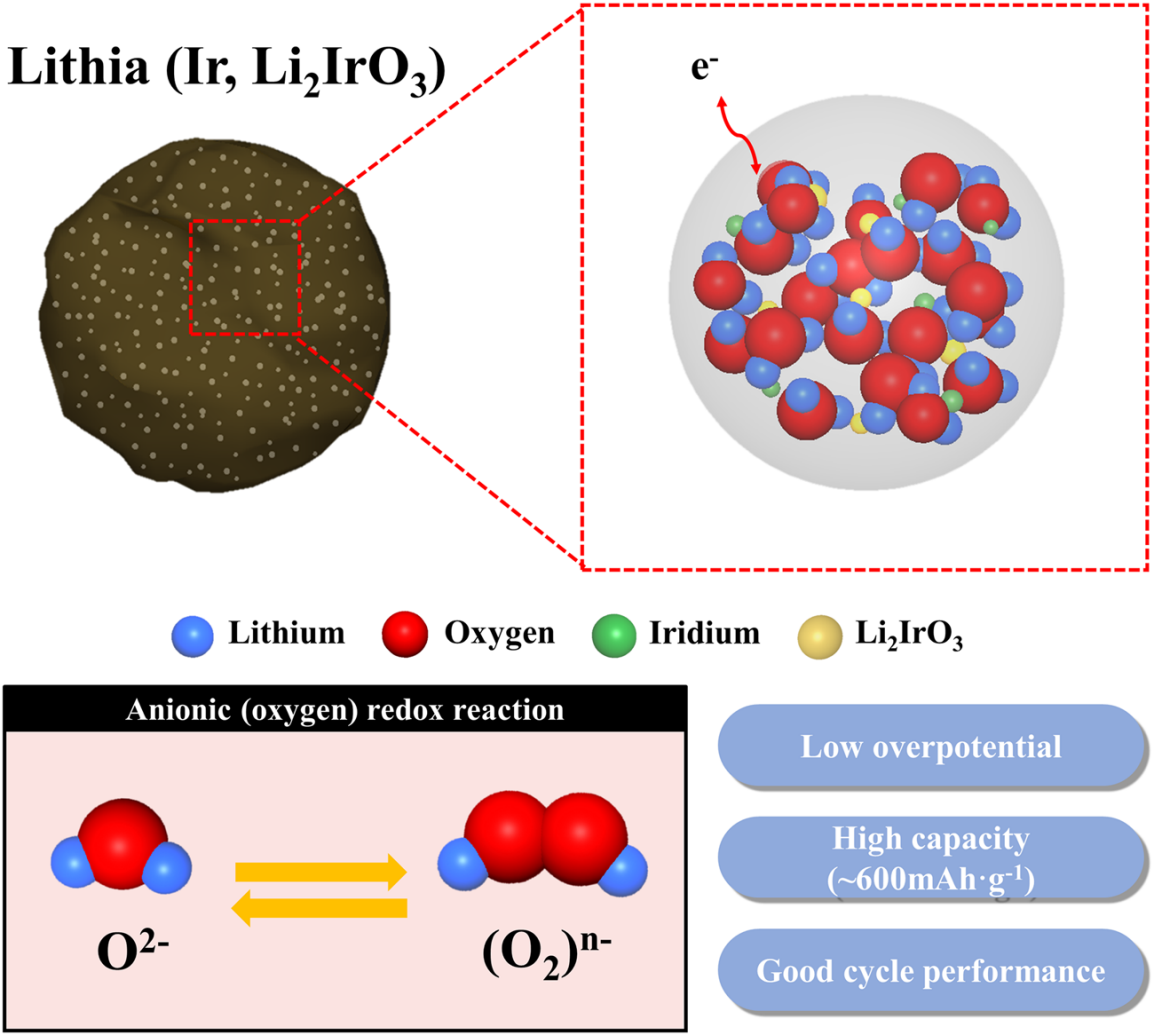
Schematic diagram of the lithia/(Ir, Li_2_IrO_3_) nanocomposite properties.

Lithia-based nanocomposites prepared using Li_2_IrO_3_ were introduced as new cathode materials for LIBs. Two types of nanocomposites were compared to determine the superior Li_2_IrO_3_ as a catalyst for nanolithia. The B-nanocomposite presented a better cyclic performance and lower overpotential compared with those of the A-nanocomposite limited capacities (300–500 mAh·g^−1^). This means that the use of 2-step Li_2_IrO_3_ is more effective for activating the capacity of nanolithia and stabilizing reaction products such as Li_2_O_2_ during cycling than the use of 1-step Li_2_IrO_3_. It is believed that the Ir-based materials formed from 2-step Li_2_IrO_3_ act as superior catalysts than those formed from 1-step Li_2_IrO_3_. The B-nanocomposite showed a stable capacity and cyclic performance at a limited capacity of 600 mAh·g^−1^. Figure 7 summarizes the properties of the lithia/(Ir, Li_2_IrO_3_) nanocomposites. XPS analysis of the electrode containing the B-nanocomposite confirmed the reversible formation and decomposition of peroxo-like (O_2_)^*n*−^ species. In contrast, the Ir oxidation state did not noticeably change. This result implies that the discharge capacity of the B-nanocomposite arises from anionic redox reactions related to oxygen rather than from cationic redox reactions. The oxygen ions in the lithia may contribute to a majority of the capacity of the nanocomposite. However, some portion of the capacity may be attributable to oxygen redox in the amorphous Li_2_IrO_3_ structure or Li-O compounds formed from the decomposition of Li_2_IrO_3_ during the milling process.

## Methods

Two types of Li_2_IrO_3_ were fabricated as catalysts for nanolithia activation. To form Li_2_IrO_3_ by 1-step heat treatment, pellets composed of IrO_2_ (AlfaAesar, 99%) and Li_2_CO_3_ (Aldrich, 99.99%) were prepared at a 1: 1.2 (wt%) ratio and calcined at 950 °C for 10 h in air. Then the calcined pellets were pulverized into powder form. This Li_2_IrO_3_ was named as ‘1-step Li_2_IrO_3_’. The other types Li_2_IrO_3_ was prepared though 2-step heat-treatment process. The calcined pellets were further (second step) heat-treated at 1000 °C for 86 h under an O_2_ atmosphere, and ground into powder. This Li_2_IrO_3_ was named as ‘2-step Li_2_IrO_3_’. The obtained two types of Li_2_IrO_3_ were dispersed in butanol (Aldrich, anhydrous, 99.8%) with lithia powder (Li_2_O, AlfaAesar, 99.5%). Ir content (f_Ir_ = Ir/(Ir + Li)) was adjusted to 0.09. Then, each Li_2_IrO_3_/Li_2_O solution was diffused via ultrasonic treatment for 30 min, and the obtained mixture was filtered and dried under vacuum at 90 °C for 24 h. To prepare lithia/(Ir, Li_2_IrO_3_) nanocomposites, the powders were mixed using planetary mono mill (Pulverisette 6, FRITSCH) at 600 rpm. The process of resting for 30 minutes after milling for one hour was repeated 150 times. Zirconia balls having diameters of 5 mm and 10 mm were used in a ratio of 1: 1 (wt%). All milling process was carried out in an Ar atmosphere using a glove box and a sealed zirconia container. XRD patterns of the synthesized powders were obtained using a Rigaku Miniflex II X-ray diffractometer over a 2θ range of 10–90° with monochromatized Cu-K_α_ radiation (λ = 1.5406 Å). To observe the shapes of the nanocomposites, transmission electron microscopy (TEM, JEM-2100F (HR)) was employed.

For electrochemical tests, the prepared nanocomposites were mixed with 30 wt% carbon nanotubes and 10 wt% polyvinylidene fluoride binder by ball-milling for 90 min. Then, the mixture was cast on aluminium foil to a thickness of 12 μm and dried under vacuum at 80 °C for 24 h. Coin cells (2032-type) were used for the electrochemical tests with Li metal as the anode, 1 M LiPF_6_ in ethylene carbonate and dimethyl carbonate (1: 1 v/v) containing 5 vol% vinylene carbonate (VC) as the electrolyte, and polypropylene (Celgard 2400) as the separator; the cells were assembled in an Ar-filled glove box. VC has been reported as an effective additive for enhancing the electrochemical performance of the lithia-based cathodes^28^. The cells were cycled in a potential range of 1.8–4.35 V with various current densities (10, 50, and 100 mA·g^−1^). The capacity of the cathode, calculated based on the lithia weight, was limited to 300–700 mAh·g^−1^. XPS (Thermo Scientific K-Alpha) was employed to elucidate the redox reaction mechanisms of the nanocomposite powders. The electrodes containing nanocomposites were collected after charging and discharging, then washed several times with dimethyl carbonate and dried under vacuum for 24 h to prepare specimens for XPS analysis.

## Supplementary information

Supplementary information
