# Supplementary material for: Lithia/(Ir, Li2IrO3) nanocomposites for new cathode materials based on pure anionic redox reaction
Source: Sci Rep. 2019 Sep 12;9:13180. doi: 10.1038/s41598-019-49806-6 (PMC6742652; doi:10.1038/s41598-019-49806-6)
Supplement: Supplementary file 1 — Supplementary information [file 41598_2019_49806_MOESM1_ESM.pdf]

Lithia/(Ir, Li_2IrO_3) nanocomposites for new cathode materials based on pure anionic redox reaction

# : Supporting Informations

Si Yeol Lee and Yong Joon Park*

Department of Advanced Materials Engineering, Kyonggi University, 154-42, Gwanggyosan-Ro, Yeongtong-Gu, Suwon-Si, Gyeonggi-Do, 16227, Republic of Korea

*Corresponding author

Ph: +82-31-249-9769; E-mail: yjpark2006@kyonggi.ac.kr

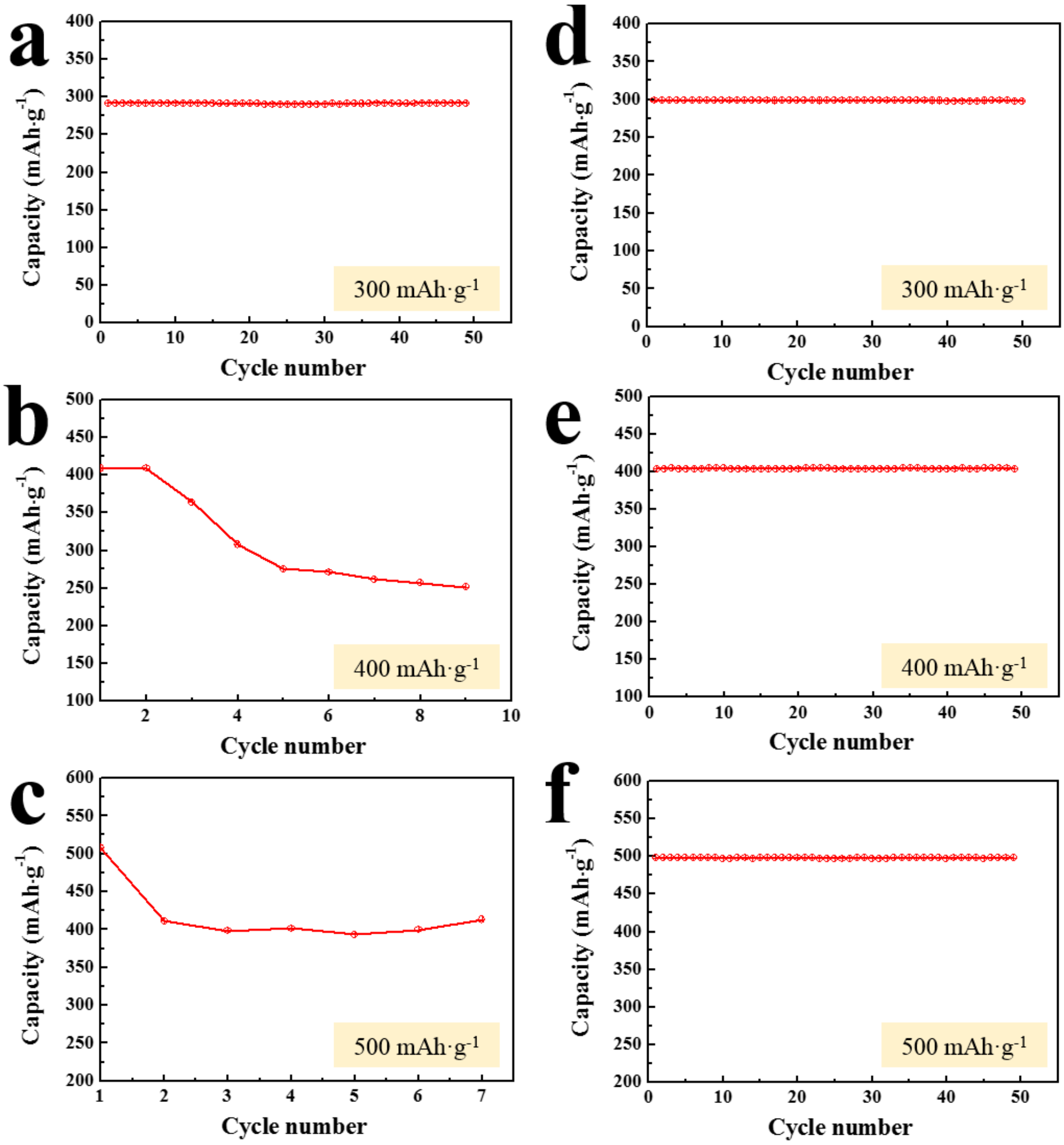

Figure S1 | Cyclic performance of nanocomposites at a current density of 10 mA·g^{-1}. (a-c) A-nanocomposite, capacity limited to 300, 400 and 500 mAh·g^{-1}, respectively; (d-f) B-nanocomposite, capacity limited to 300, 400 and 500 mAh·g^{-1}, respectively.

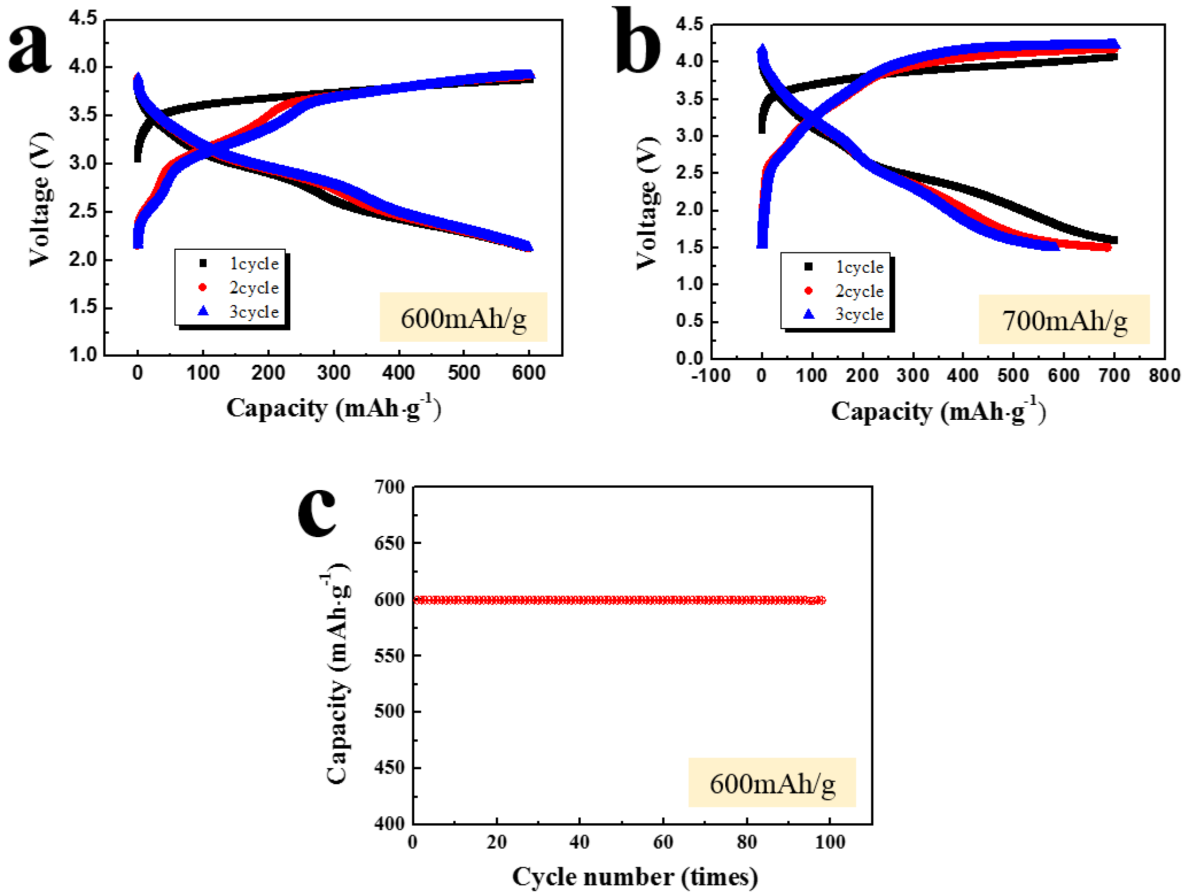

Figure S2 | Voltage profiles and cyclic performance of the B-nanocomposite at a current density of 50 mA·g^{-1}. (a) Voltage profile at limited capacity of 600 mAh·g^{-1} and (b) 700 mAh·g^{-1}; (c) cyclic performance at limited capacity of 600 mAh·g^{-1}.

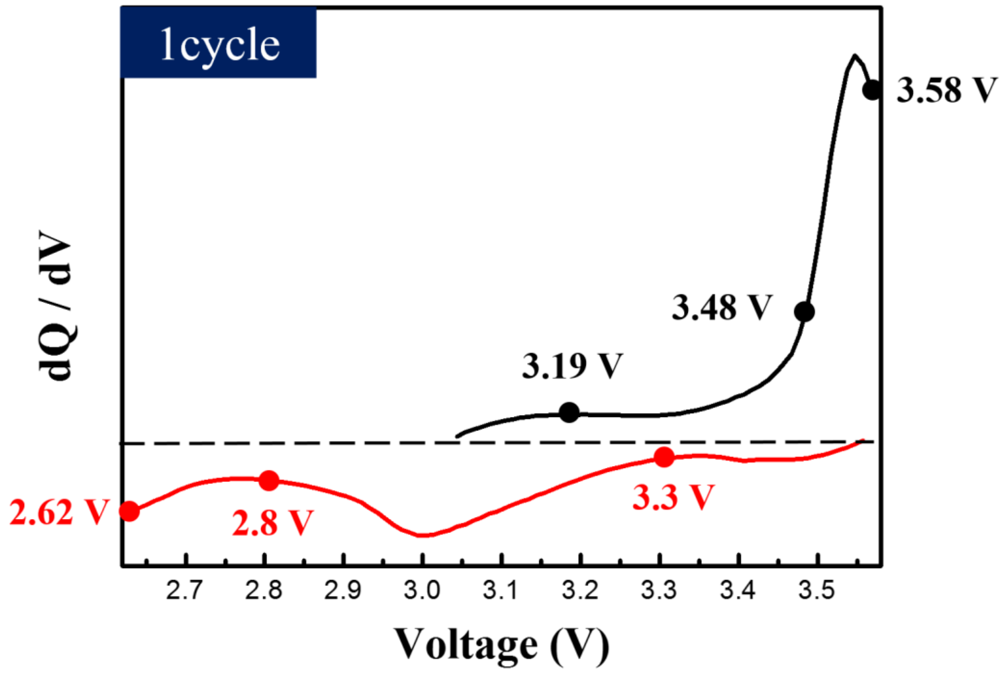

Figure S3 | Corresponding derivative plots dQ/dV for the initial cycle of the B-nanocomposite (capacity limited to 500 mAh·g^{-1}).
